# Effect of Dextrose Equivalent on Maltodextrin/Whey Protein Spray-Dried Powder Microcapsules and Dynamic Release of Loaded Flavor during Storage and Powder Rehydration

**DOI:** 10.3390/foods9121878

**Published:** 2020-12-17

**Authors:** Kaixin Li, Bowen Pan, Lingjun Ma, Song Miao, Junfu Ji

**Affiliations:** 1 Key Lab of Fruit and Vegetable Processing, National Engineering Research Center for Fruit and Vegetable Processing, Ministry of Agriculture and Rural Affairs, College of Food Science and Nutritional Engineering, China Agricultural University, Beijing 100083, China; likx_1108@163.com (K.L.); panbowen19950728@163.com (B.P.); lingjun.ma@cau.edu.cn (L.M.); 2 Xinghua Industrial Research Centre for Food Science and Human Health, China Agricultural University, Xinghua 225700, China; 3 Teagasc Food Research Centre, Moorepark, Fermoy, R93 XE12 Co. Cork, Ireland; song.miao@teagasc.ie

**Keywords:** maltodextrin, powdered microcapsules, dynamic flavor release, solubility, stability

## Abstract

The preparation of powdered microcapsules of flavor substances should not only protect these substances from volatilization during storage but also improve their diffusion during use. This study aimed to investigate the effects of maltodextrin (MD) with different dextrose equivalent (DE) values on retention of flavor substances during storage, and the dynamic release of flavor substances during dissolution. MDs with three different DE values and whey protein isolate were mixed in a ratio of 4:1 as wall materials to encapsulate ethyl acetate, and powdered microcapsules were prepared by spray drying. It was proved that MD could reduce the diffusion of flavor substances under different relative humidity conditions through the interaction between core material and wall material. During dissolution, MD released flavor substances quickly owing to its superior solubility. The reconstituted emulsion formed after the powder dissolved in water recaptured flavor substances and made the system reach equilibrium. This study explored the mechanism of flavor release during the storage and dissolution of powder microcapsules and should help us understand the application of powder microcapsules in food systems.

## 1. Introduction

Flavor is an indispensable feature of food, and the effective use of the characteristics of flavor substances can increase the quality of food. However, flavor substances have low molecular weights and easily volatilize under the influence of variations in humidity, temperature, and light intensity [1]. In addition, most volatile flavor substances have a strong affinity for lipids and are preferentially soluble in oil phases, which limits their applicability in aqueous food matrices and may cause the delayed or incomplete release of these flavor substances [2]. Therefore, encapsulation is an effective technique for the design of suitable structures for protecting flavor substances from damage due to the external environment during storage, as well as accelerating flavor release upon consumption. At present, various emulsions are used as liquid encapsulation materials, but most of these are thermodynamically unstable systems. Many studies have proved that sensitive flavor substances can be effectively encapsulated in powered microcapsules by natural or synthetic polymer materials. In comparison with liquid emulsions, powder microcapsules have significantly higher stability and are more convenient in terms of transportation and storage. However, rapid rehydration is necessary for these powders, as it is a prerequisite for them to achieve their functionalities upon consumption. Consequently, wall materials are believed to play critical roles in improving the functionalities of core flavor substances. The encapsulation process involves encapsulating the sensitive substances in natural or synthetic polymer materials by forming a continuous film, which is coated on the surface of the core material in the form of tiny droplets or particles, and finally preparing microcapsules [3]. Thus, it is necessary to devise a suitable system for encapsulating flavor substances in a food system.

Food proteins are often used as emulsifiers in encapsulation because of their amphiphilic properties. Whey protein isolate (WPI), of which β-lactoglobulin is the main component, plays an important role in combining with flavor substances owing to the existence in this molecule of two independent binding sites [4]. Previous studies have shown that wall materials with hydrophobic cavities can combine with flavor substances via noncovalent bonds and thus improve the oxidation stability of core materials, and the release behavior during storage depends on the strength of these bonds [5]. However, the use of protein alone as the wall material to encapsulate lipids is likely to cause oil droplets to spread out during drying. Moreover, the addition of a carbohydrate to a protein matrix can prevent the outward diffusion of hydrophobic core materials and also improve the oxidation stability of lipids during storage [6].

Some natural polysaccharides have properties such as high viscosity and poor solubility that limit their applicability in spray drying [7]. Maltodextrin (MD) is a starch hydrolysis product with a dextrose equivalent (DE) of less than 20 and is widely used in microencapsulation because of its high solubility and ability to form low-viscosity solutions at high concentrations [8]. The DE value of MD varies with changes in the chain lengths of sugar molecules. The shorter is the average chain length, the higher is the DE value. Moreover, MDs with different DE values have different properties and functions in microcapsules. Matsuura et al. [9] used MDs with DE values of 2, 10, and 25 as wall materials because of differences in the composition and structure of MD and in its interactions with emulsifiers. Although MD with a DE value of 10 had the strongest effects with emulsifiers, the powder formed by spray drying had the lowest stability. However, high DE values can improve the stability of the core materials in the spray drying process because the matrices are more uniform after drying, which thereby increases the retention rate and produces a powder with low hygroscopicity [10]. Further, the solubility of MD increases with an increase in the DE value, which may accelerate the dissolution of powder microcapsules and the release of flavor. Therefore, we should pay more attention to understanding the properties of the wall material with regard to the dynamic release of flavor substances during storage and the dissolution process, which will be helpful for understanding its application in real food systems.

In this study, the volatile ethyl acetate (EA) was selected as a model core substance. WPI mixed with MDs with different DE values was used as the wall material to create powder microcapsules by spray drying. The effects of MDs with different DE values on the stability of EA during storage were investigated in terms of dynamic flavor release and structural changes in the wall materials. In addition, the release behavior of EA during rehydration of the powder was studied. It is hoped that this study will provide more ideas for controlling the release of flavor from powder microcapsules in food systems during storage and consumption.

## 2. Materials and Methods

### 2.1. Materials and Reagents

WPI containing 89% protein, 0.5% fat, and 4.4% moisture was purchased from Ingredia Dairy Experts (Wapakoneta, OH, USA). Glucidex 6 (MD with a DE value of 6, denoted as MD 6), Glucidex 12 (MD with a DE value of 12, denoted as MD 12), and Glucidex 17 (MD with a DE value of 17, denoted as MD 17) were all purchased from Sigma-Aldrich (St. Louis, MO, USA). EA (>99% purity), hydroxylamine hydrochloride, and ethanol (>99.7% purity) were offered by Sinopharm Chemical Reagent Ltd. (Shanghai, China), and *n*-hexane was purchased from Aladdin Industrial Corporation (Shanghai, China). Protease (~200 U/mg) was obtained from Beijing Solarbio Science & Technology Ltd. (Beijing, China).

### 2.2. Preparation of Encapsulation Precursors

MD 6, MD 12, and MD 17 were mixed with WPI powder in a ratio of 4:1 (*w*/*w*) and stirred at 1000 rpm for 2 h at 40 °C to prepare the wall materials. The core material was formed from a mixture of EA and soybean oil in a ratio of 2:1 (*w*/*w*), which was then slowly poured into the wall material solutions in a water-to-oil ratio of 10:1 (*w*/*w*). The resulting coarse emulsions were treated with a high-speed homogenizer (JN-10HC, Guangzhou Juneng Nano&Bio Technology Ltd., Guangzhou, China) at 10,000 rpm for 5 min and were further homogenized at 50 MPa twice at room temperature. Finally, the zeta-potentials and average particle sizes of the emulsions were measured by a light scattering instrument (Zetasizer Nano-ZS90, Malvern Instruments Ltd., Worcestershire, UK).

### 2.3. Spray Drying System

The liquid emulsions were spray-dried with a mini spray dryer (B-290, Büchi Corporation, Flawil, Switzerland) to produce powder microcapsules. The inlet air and outlet air drying temperatures were set at 135 °C and 70 °C, respectively. The pump speed was set at 10.5 mL/min, and the air flow rate was set as 0.667 m^3^/h. Samples containing WPI, WPI/MD (DE 6), WPI/MD (DE 12), and WPI/MD (DE 17) as the wall material were denoted as P_0_, P_1_, P_2_, and P_3_, respectively. The dried samples were kept in desiccators with P_2_O_5_ until further analysis. 

### 2.4. Physical Properties of Powder

#### 2.4.1. Moisture Content

The moisture content of powder microcapsules was measured by the method (AOAC, 2006) [11]. Samples of about 3 g were weighed and dried at 105 ± 1 °C for 3 h until they reached a constant weight (M_1_). The moisture content (MC) of the powder was calculated by the following Equation (1), where M_0_ is the original weight of a sample:

(1) $$\mathrm{MC}\left(\%\right)=\frac{M_{0}-M_{1}}{M_{0}}\times 100$$

#### 2.4.2. Density and Flowability

Powder samples of approximately 1.5 g were put in a tapped machine (HY-100, Haoyu Technology Co., Ltd., Liaoning, China), and then the surface was gently flattened for the measurement of the powder volume (V_b_). The bulk density (ρ_b_) of the powder sample was calculated by the following Equation (2), where m is the weight of the powder sample:

(2) $$\rho_{b}=\frac{m}{V_{b}}$$

The measuring cylinder was then placed on a tap density tester, and the vibration frequency was set to 1250 min^−1^ until the height of the powder sample no longer changed. The volume of the powder (V_t_) was recorded and used to calculate the tap density (ρ_t_) of the powder sample by the following Equation (3):

(3) $$\rho_{t}=\frac{m}{V_{t}}$$

The ability of a powder to flow freely in a regular and constant manner is called flowability. The Hausner ratio (HR) and Carr’s compressibility index (CI) are generally used for the analysis of powder flowability. It is calculated by the numerical value of bulk density and tap density, as shown in the following Equations (4) and (5):

(4) $$\mathrm{HR}=\frac{\rho_{t}}{\rho_{b}}$$

(5) $$\;\mathrm{CI}\left(\%\right)=\frac{\rho_{t}-\rho_{b}}{\rho_{t}}\times 100\;$$

#### 2.4.3. Encapsulation Efficiency (EE)

Ultraviolet-visible spectrophotometry was used to determine the EA content in powder microcapsules. In brief, 0.20 g powder was dissolved in 20 mL distilled water and mixed with 0.10 g neutral protease. The mixture was added to 10 mL *n*-hexane, treated on a high-speed shearing machine at 10,000 rpm for 1 min, and then incubated in a water bath at 45 °C for 15 min. After that, the samples were cooled to room temperature and centrifuged at 5000 rpm for 10 min to segregate EA from the aqueous phase. A chromogenic agent was added to the supernatant for determination by a spectrophotometer using the absorbance at 525 nm. The amount of EA was quantified from the standard calibration curve and was denoted as TO. The loading capacity of powder was determined as following Equation (6):

(6) $$\mathrm{Loading}=\frac{\mathrm{EA}\;\left(\mathrm{mg}\right)}{\mathrm{Powder}\;\left(g\right)}$$

The EA content on the powder surface was measured as described previously [12]. A powder sample of 0.2 g was first mixed with 10 mL *n*-hexane and then sealed and shaken well to extract the surface oil within 2 min at room temperature. Then 0.5 mL of the supernatant was taken for the further measurement of the EA content on the powder surface, which was denoted as SO. Therefore, the EE of the powder could be calculated by the following Equation (7):

(7) $$\mathrm{EE}\left(\%\right)=\frac{\mathrm{TO}-\mathrm{SO}}{\mathrm{TO}}\times 100$$

#### 2.4.4. Particle Size and Microscopic Morphology

The microstructure of the powder was observed by a scanning electron microscope (Zeiss Supra 55 Gemini; Carl Zeiss, Jena, Germany). A small amount of powder was taken and pasted onto a conductive tape. Then samples were analyzed by the scanning electron microscope operating at an accelerating voltage of 25 kV. Representative micrographs were selected for presentation. The particle size of powder samples was measured by a light scattering instrument (Zetasizer ZEN 3700, Malvern Instruments Ltd., Worcestershire, UK).

### 2.5. Flavor Release during Storage

The dynamic flavor release from powder microcapsules was investigated under conditions of different relative humidity (RH), which imitated storage environments. All powders continued to be dried in the vacuum oven together at 35 °C to obtain the final moisture content of about 1.1 ± 0.08%. Powder samples of 1 g were weighed and added to 250 mL headspace bottles, which were charged with 4 mL saturated solutions of CH_3_COOK, K_2_CO_3_, NaNO_2_, and NaCl at 25 °C to give different hygroscopic environments with RH values of 22%, 43%, 65%, and 75%, respectively. The headspace bottles were sealed, incubated in vacuum desiccators, and sampled at time points of 3, 6, 12, 24, 48, 72, and 120 h. The retention of EA in the powder microcapsules was measured by the method described in Section 2.4.3.

In addition, the headspace concentration of EA was measured by gas chromatography–mass spectrometry (GC-MS) [13]. A sample of 1 mL headspace air was taken with an airtight needle and then injected into a gas chromatograph. Helium (>99.99% purity) was used as the carrier gas at a flow rate of 0.8 mL/min. The injector temperature was set at 260 °C, and the oven temperature was initially set at 50 °C for 5 min and then raised to 180 °C at a rate of 10 °C/min. The peak area for EA was integrated and used to determine the release intensity of EA.

### 2.6. Hygroscopicity of Powder Microcapsules

The hygroscopicity of the powder during storage was quantified by the gravimetric method. The mass of a 1 g powder sample was recorded as the initial mass, and the sample was stored in a glass vial of known mass under the same storage conditions as described in Section 2.5. Changes in the sample weight were measured at time points of 3, 6, 12, 24, 48, 72, and 120 h during storage. The measurement of each sample is controlled within 20 s. The increase in the mass of the powder system was assumed to be due to adsorbed moisture, and the average weight of triplicate samples was used in calculations.

### 2.7. Characterization of Changes in Structure of Powder Microcapsules during Storage

#### 2.7.1. Fourier Transform Infrared Spectroscopy (FTIR)

A powder sample was mixed with dried KBr in a ratio of 1:100 and ground thoroughly to form a tablet. Spectra were recorded over the range from 400 cm^−1^ to 4000 cm^−1^ with a resolution of 4 cm^−1^. The dried KBr was used as a control, and the sample was scanned by a Tensor 27 Fourier infrared spectrometer. 

#### 2.7.2. Fluorescence Spectroscopy

A 50 mg powder sample was dissolved in 10 mL phosphate-buffered saline (PBS) solution (pH 7.6) and added to a quartz cuvette. The sample was scanned by an F-7000 FL spectrophotometer, and the operational parameters of fluorescence spectroscopy were as follows: the excitation wavelength was set at 275 nm; the slit widths for both excitation and emission were set at 5 nm; and the emission wavelength ranged from 290 to 450 nm. The PBS background spectrum was finally subtracted from the sample spectrum. The emission spectrum of the sample was recorded in the range from 300 to 400 nm. For each emission wavelength, the result was the sum of three scans.

#### 2.7.3. X-ray Diffraction Analysis (XRD)

A small amount of powder microcapsules were taken for X-ray diffraction analysis. X-ray diffraction patterns were obtained by diffractometer system (Bruker D8 Venture, Bruker Ltd., Hamburg, Germany). The scanning range was set to 5–30° and the operating electric voltage and current were adjusted at 40 kV and 30 mA, respectively. A total of three repetitions of each sample were examined.

#### 2.7.4. Confocal Laser Scanning Microscopy (CLSM)

The powder microstructure was imaged by CLSM. A few samples were taken and scattered on a glass slide, and solutions of Fast Green (1 mg/mL in water) and Nile red (0.1 mg/mL in ethanol) were mixed and dropped onto the powder samples for 1 min. Finally, the samples were covered with a slide and imaged under a microscope with a 100× oil immersion objective. The emission filters were set at 633 nm for Fast Green and 488 nm for Nile red, which were used to distinguish protein and oil phases, respectively. 

### 2.8. Characterization of the Dissolution Process of Powder Microcapsules

#### 2.8.1. Dynamic Dissolution Process

A 2 g powder sample was dissolved in 50 mL deionized water and stirred with a magnetic stirrer at a speed of 100 rpm at 25 °C. A 15 mL sample of the resulting suspension was taken at the same depth in the beaker each time, and the sampling time points were set at 1, 5, 10, 15, 30, 45, 60, and 90 min during dissolution. The suspension was centrifuged at 4000× *g* for 10 min, and the 10 mL supernatant was separated and moved to an oven at 105 °C for 6 h with the aim of reaching a constant weight for measuring the solid content in the solution.

#### 2.8.2. Flavor Release during Dissolution

A 2 g powder sample and 50 mL water were mixed in a headspace bottle. The dissolution process was carried out by magnetic stirring in a closed environment. At time points of 1, 5, 10, 15, 30, 45, 60, and 90 min during dissolution, 1.0 mL headspace gas was extracted to determine the concentration of EA, which was released into the headspace bottle corresponding to the extent of dynamic dissolution. The method was the same as that mentioned above in Section 2.5. Moreover, the amount of EA remaining in the liquid phase was also determined according to the method mentioned above in Section 2.4.3.

### 2.9. Statistical Analysis

All samples were carried out in triplicate. Mean deta of water sorption, density and flowability, encapsulation efficiency, particle size, flavor retention and release, FTIR, fluorescence spectroscopy, XRD were repeated at least three times, and the results reported are the average values of measurements. The significance level was set at *p* < 0.05. Analysis of variance was performed by SPSS version 17.0 (SPSS, Inc., Chicago, IL, USA).

## 3. Results and Discussion

### 3.1. Characterization of Microcapsules

#### 3.1.1. Physicochemical Properties

The effects of MDs with different DE values on the properties of EA microcapsules are presented in Table 1. Protein is an effective carrier agent for spray drying, which can form a thin film in the drying process and obtain high yield. The addition of MD changed the viscosity of the emulsion, and reduced the protein content in the system, resulting in a reduction in the yield. The moisture contents of the four samples were all less than 5%, and those of the samples in which MD was used as the wall material were significantly lower (*p <* 0.05) than that of the control sample, which conforms to the requirement for the maximum moisture content of a food powder (4–6%) and is suitable for long-term storage [14]. When MD with a DE value of 17 was used, the water content of the sample was the lowest, namely, 4.16%. The particle sizes of all the samples were about 9 μm, which was characteristic of the sizes of particles obtained by spray drying [15]. In addition, Table 1 shows that there was no significant difference between the tap densities. The bulk density of the control sample was slightly higher, which might have been due to a higher protein content [16]. The dense structure of whey protein also caused significantly poorer powder flowability.

The EE values of the four samples ranged from 70% to 85%, among which P_3_ had the highest EE, namely, 84.23%. In addition to the EE, the loaded amount of the core material is also an important indicator for determining the embedding effect in microcapsules. The loaded amounts of EA in the four samples were different and ranged from 13.93 mg/g to 19.83 mg/g. The loading capacity of P_0_ was the highest, whereas that of P_3_ was the lowest (*p <* 0.05). It is worth noting that in the samples containing MD, the EE increased with an increase in the DE value, which is consistent with previous studies. MD can form a hydrophobic region in an aqueous solution and capture hydrophobic molecules via hydrophobic interactions with flavor substances [17]. With an increase in the DE value, more hydrophobic areas are exposed and the interaction with EA is enhanced, which thus increases the EE. In addition, Hogan et al. [18] have shown that a high DE value can minimize the loss of oil droplets during spray drying and thus effectively increase the EE. This may explain why MD with a high DE value had a high EE.

#### 3.1.2. Morphology of Powder Microcapsules

**Figure 1:**
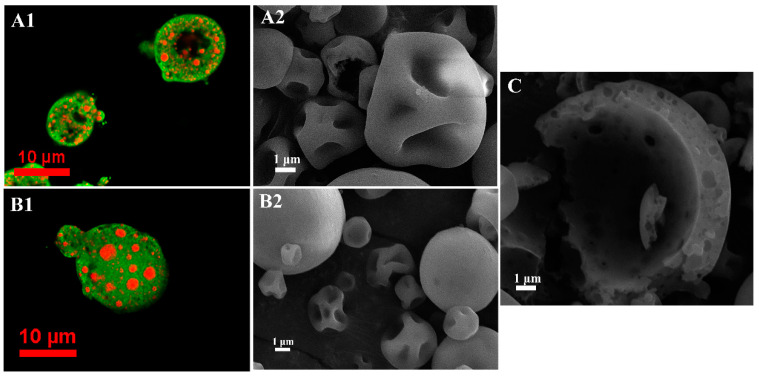
SEM (10,000×) and CLSM (100×) images of powdered microcapsules. ((**A1**) and (**A2**) whey protein isolate (WPI) used as wall materials alone, (**B1**) and (**B2**) WPI and maltodextrin (MD) with dextrose equivalent (DE) values of 18 used as wall materials, (**C**) inside of powdered microcapsules).

As shown in Figure 1, the morphology of powder microcapsules was spherical with a smooth surface and no obvious cracks. Figure 1A1,B1 obtained using CLSM show oil droplets of EA (red) in the wall materials (green), which implies that the flavor substance was successfully encapsulated in the powder microcapsules. However, the deformation and concave and irregular surfaces of powder samples were caused by the rapid loss of water and shrinkage of the particle surfaces during spray drying [19]. The surface morphology of P_3_ was comparatively smoother (Figure 1B2), as MD with a high DE value may act as a plasticizer to prevent the irregular shrinkage of spherical particles [10]. It may be considered that the degree of concavity of the powder was correlated with the content of oil droplets on the surface, and a higher degree of concavity led to a higher content of free oil droplets [20]. This may also explain why P_3_ had the highest EE. In addition, if the powder microcapsules were cut, an internal hollow structure could be observed and the porous shell wall was perforated by small holes (Figure 1C). It is believed that the core materials are generally embedded in the pores of spherical powder particles after drying, and this kind of structure is also an important indicator for controlling the release of the core materials [21]. 

### 3.2. Hygroscopicity of Microcapsules during Storage

**Figure 2:**
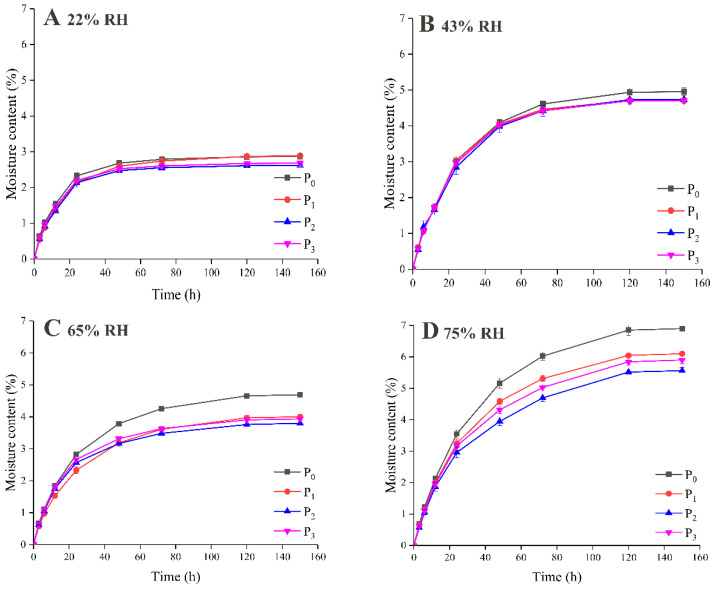
Water adsorption of powdered microcapsules at relative humidity (RH) 22% (**A**), 43% (**B**), 65% (**C**), 75% (**D**) during storage. The wall material was WPI (P_0_), WPI/MD (DE 6) (P_1_), WPI/MD (DE 12) (P_2_), WPI/MD (DE 17) (P_3_).

The hygroscopicity of powder microcapsules during storage is shown in Figure 2. In general, the adsorbed water content of the powders increased with increases in the storage time and environmental RH. All the samples took up moisture quickly in the first 12 h, and then slowly increased until equilibrium was reached at 150 h. Furthermore, as shown in Figure 2A,B, the difference between samples was not significant if they were stored in an environment with a low RH ranging from 22% to 45%. The adsorbed water content after 120 h was close to 3% and 5%, respectively. However, when the RH reached 65%, the adsorbed water content of P_0_ was significantly higher than those of the samples with added MD. This phenomenon was more obvious when the RH reached 75%, when the moisture content of P_0_ was nearly 7%, which was much higher than those of the samples that contained MD. 

In the case of spray-dried protein powder, an increase of RH will increase the water adsorption capacity of the powder. Meanwhile, MDs with low DE values have longer molecular chains and more hydroxyl functional groups, which can be used to adsorb more water. Castro et al. [22] found that MD with a DE value of 6 has the strongest hygroscopicity, which corresponds to the finding that P_1_ had higher hygroscopicity than P_2_ and P_3_. The water adsorption behavior of the different wall materials had a sequential relationship [23]. When the RH was low (22% and 43%), there was no significant difference in hygroscopicity among the samples, because the protein adsorbed water first and inhibited the crystallization of the polysaccharide. However, with an increase in humidity (65% and 75%), the adsorbed water contents of the samples with added MD decreased in comparison with that of the sample containing WPI alone. In addition, in comparison with polysaccharides, WPI has stronger hygroscopicity and is conducive to the adsorption of water molecules [24]. The addition of MD also reduced the content of WPI, and hence the hygroscopicity of P_0_ was higher than those of P_1_, P_2_, and P_3_.

### 3.3. Flavor Release during Storage

**Figure 3:**
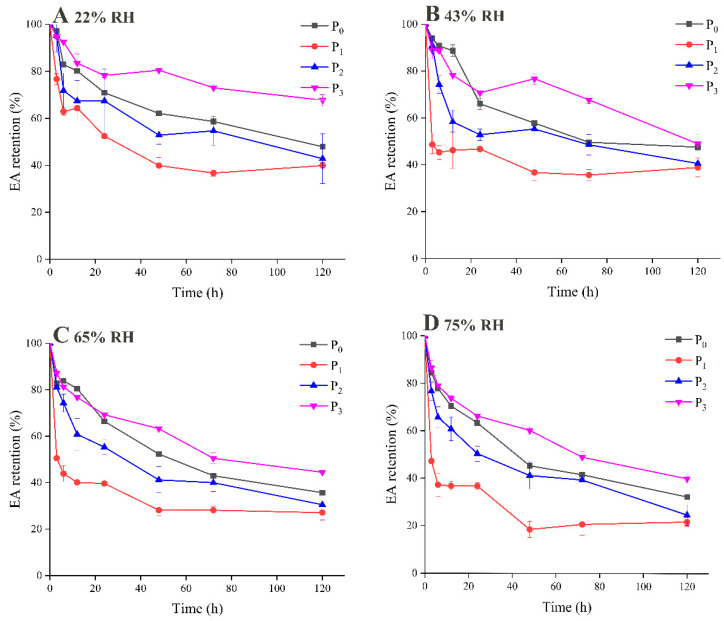
Ethyl acetate (EA) retention of powdered microcapsules at relative humidity (RH) 22% (**A**), 43% (**B**), 65% (**C**), 75% (**D**) during storage.

**Figure 4:**
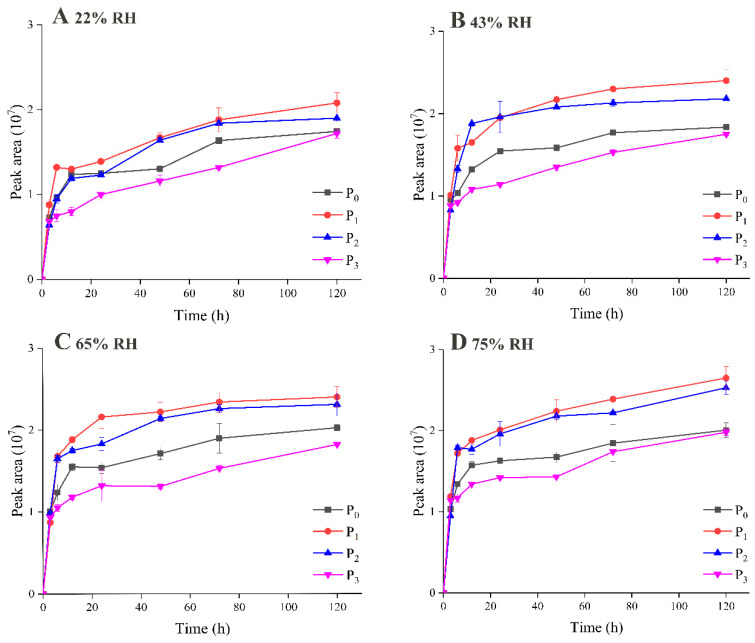
Ethyl acetate (EA) release of powder microcapsules at relative humidity (RH) 22% (**A**), 43% (**B**), 65% (**C**), 75% (**D**) during storage.

The dynamic flavor release from powder microcapsules within 120 h was investigated, as illustrated in Figure 3 and Figure 4, which show the results for the retention and release of EA during storage, respectively. It is not surprising to see that the retention of EA in all the samples decreased dramatically during the whole storage period, as volatile EA is not stable at room temperature and is prone to escape from a food matrix. The samples in which MD was used as the wall material displayed completely different flavor release behaviors, depending on the DE value. When those samples are compared, it is clearly observed that P_3_ exhibited significantly greater retention ability for EA during storage. After 120 h, P_3_ retained more than 70% of its flavor substance content at an RH of 22%, and, even at an RH of 75%, 40% of the EA content was still encapsulated by the wall material, which consisted of MD with a high DE value and whey protein. In addition, the flavor retention abilities of P_1_ and P_2_, which had low and medium DE values, were significantly lower than those of the other samples, including the control sample P_0_. Only about 20% of the EA content remained at the end of storage at an RH of 75%.

The same trend was also seen and confirmed by using GC-MS to measure the headspace concentration of EA during storage, as shown in Figure 4. The released amounts of EA were similar at a lower RH, which indicated that the powders remained relatively stable in this environment. However, at an RH of 75% the released amounts for MDs with low and medium DE values increased significantly, and the released amount for P_1_ was 1.4 times as much as that for P_3_. 

The content of EA in all the samples decreased rapidly in the first 12 h, which may have been caused by two factors. Firstly, EA on the surfaces of the microcapsules rapidly dissipated. On the other hand, the adsorption of water by the microcapsules led to the destruction of the powder structure, the amorphous MD may change from glassy state to rubber-like state. Therefore, the internal oil droplets accumulated and migrated outward, which resulted in rapid flavor release. However, the DE value of MD did not correspond to the surface flavor dissipation rate but significantly affected the flavor release rate inside the microcapsules [10]. The retention of EA decreased slowly during the storage period from 20 h to 120 h, which indicated that encapsulation in microcapsules effectively delayed the release of EA under the same storage conditions. MD with a high DE value (P_3_) has a better retention effect on flavor compounds. On the one hand, it has greater adhesive strength and stability, and more easily forms a glassy matrix. Adding MD to the protein system can increase the glass transition temperature of the mixed system, and exhibits stronger resistance to a humid environment during storage, thus the flavor retention rate is increased, which is similar to previous results [25]. In addition, a higher DE value of MD is beneficial for the formation of a particle barrier, which increases the resistance to permeation and protects the lipophilic materials enveloped in the interior. When MD has a low DE value, the main part of the molecule contains more long-chain sugars, which constitute a poor barrier to oxygen in storage environments and accelerate the diffusion of oil droplets of EA [26]. On the other hand, the hydrophobic region formed by WPI and MD as the core materials combined with oil droplets. The stronger was the binding force of the P_0_ and P_3_ sample, the better was the retention effect on flavor compounds.

### 3.4. Changes in Microcapsule Structure During Storage

#### 3.4.1. FTIR and Fluorescence Spectroscopy

**Figure 5:**
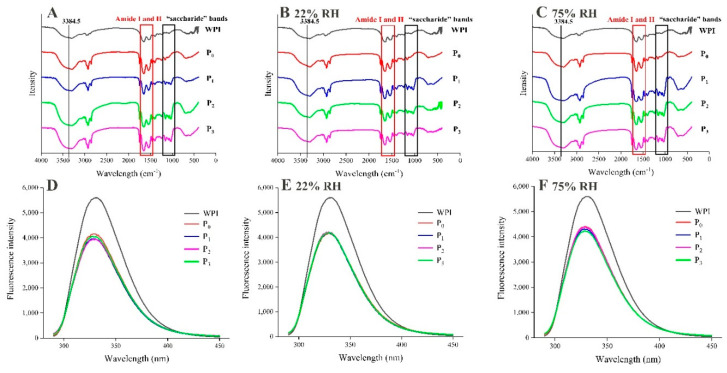
FTIR and fluorescence spectrum for WPI and powdered microcapsules of storage for 12 h. ((**A**) FTIR before storage, (**B**) FTIR at 22% RH, (**C**) FTIR at 75% RH, (**D**) Fluorescence spectrum before storage, (**E**) Fluorescence spectrum at 22% RH, (**F**) Fluorescence spectrum at 75% RH).

According to the results for flavor release during storage, it was believed that 12 h might be the inflection point in the entire release process. FTIR and fluorescence spectroscopy were therefore used to measure the structural changes in the wall materials at this time point. It can be seen from Figure 5A–C that the structure of the microcapsules was still dominated by WPI. Changes in peaks in the region of 953–1180 cm^−1^ were used to determine changes in the polysaccharide in microcapsules. These peaks are usually caused by the stretching of C-C and C-O bonds and the bending of C-H bonds [27]. After the addition of MD, the sizes of these peaks changed markedly, which indicated that using MDs with different DE values will have a certain effect on the microcapsule structure. There were three characteristic peaks due to protein, which were caused by vibrations of amide I (C=O) at 1600–1700 cm^−1^, amide II (60% N-H and 40% C-N) at 1500–1600 cm^−1^, and amide A (N-H) at 3300–3400 cm^−1^. The peak sizes for both amide I and amide II changed after microencapsulation. The absorption peak at 3384.5 cm^−1^ may have been due to the stretching vibrations of -OH groups, and the low peak wavenumber indicates that the hydrogen bonding interactions were weakened, which implies that the sample was more hydrophobic [28]. In the storage process, the peaks at an RH of 75% were lower than those at an RH of 25%, which also indicates that wall materials may combine more strongly with hydrophobic flavor substances and play a positive role in flavor retention during storage.

Similar results were obtained by fluorescence spectroscopy of the samples (Figure 5D–F). WPI gave rise to a peak at 330.6 nm, which is consistent with the previous results [13]. Different degrees of blueshift were observed for the microcapsule samples, and the fluorescence intensity was lower, which indicates that EA had combined with the hydrophobic region of the wall material. After storage for 12 h, the fluorescence intensity increased at 330 nm, especially at an RH of 75% (Figure 5F), which indicates that the adsorption of water by the microcapsules led to structural damage and the binding force between the core and wall materials was weakened.

#### 3.4.2. X-ray Diffraction of the Powder Microcapsules

**Figure 6:**
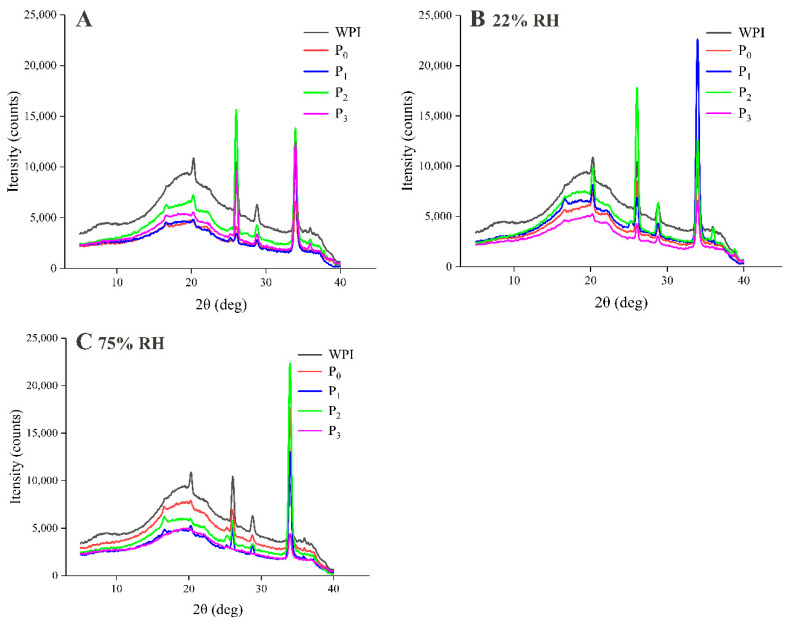
X-ray diffraction of WPI and powdered microcapsules before storage (**A**) and at relative humidity (RH) 22% (**B**), 75% (**C**) during storage of storage for 12 h.

Figure 6 shows the X-ray diffractograms of powder microcapsules at RH values of 22% and 75%. WPI gave rise to a characteristic peak at 19.66°, which is indicative of the presence of crystalline regions and noncovalent bonds [29]. In the patterns of the microcapsule samples, the broad peak shifted to lower angles, which indicates interactions between the core material and wall material (Figure 6A). During storage, it can be seen that the change in the pattern of P_3_ was the smallest, which indicates that it exhibited strong resistance to a humid environment and could better protect flavor compounds (Figure 6B,C). In contrast, the sample without MD (P_0_) was greatly affected by humidity, and the binding force between the wall material and the core material was gradually weakened, which resulted in the loss of the flavor substance. In addition, the broad peak in the pattern of P_2_ was the most similar to that of WPI over the whole storage period, which indicates that the wall material and core material had the weakest binding force and the retention of the flavor substance was the lowest, which was similar to the previous results. Therefore, it can be concluded that the tendency in flavor retention was the same as that in the results for flavor release during storage.

#### 3.4.3. Confocal Laser Scanning Microscopy (CLSM)

**Figure 7:**
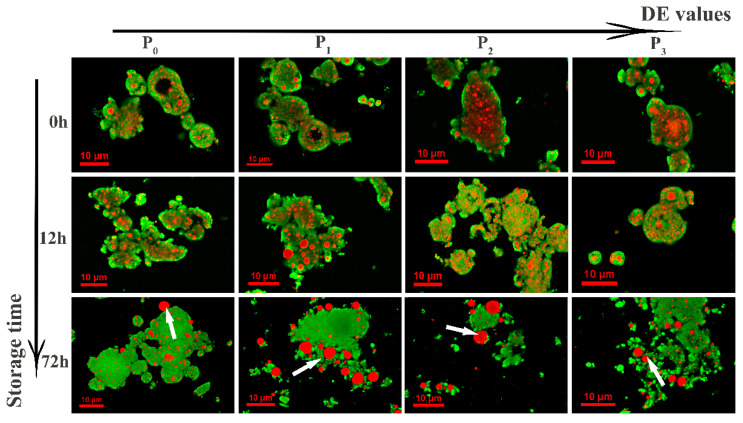
Confocal laser scanning microscopy confocal laser scanning microscopy images of powdered microcapsules after storage at 75% RH for 0 h, 12 h and 72 h. The DE values means dextrose equivalent (DE) values.

The structure of microcapsules and the distribution of EA during flavor release could be observed and analyzed by CLSM. The samples stored at an RH of 75% were selected as representative on the basis of previous results for flavor retention and headspace concentration. In Figure 7, the green color represents the protein matrix, whereas the red color indicates oil droplets of EA. At the beginning of storage (0 h), it is clearly seen that small droplets of EA were evenly distributed in the internal structure of the powder matrix, which proves that wall materials could play a useful role in the encapsulation of flavor substances. This kind of matrix structure was formed by the homogenization of protein and EA droplets, as well as the spray drying process that produced powders [30]. After 12 h, the EA droplets became prone to aggregate together and gradually spread outward, which indicated that the flavor substance was being released from the powder microcapsules. Especially in the case of P_1_, EA was distinctly assembled and formed the largest droplets, whereas there were significantly fewer droplets in P_3_, which indicated that MDs with high DE values had a better protective effect on the flavor substance. After storage for 72 h, small droplets in all the samples accumulated from the internal structure, gradually migrated to the powder surface, and finally diffused to the exterior. It can clearly be seen that in P_1_ and P_2_ the droplets were the largest and most diffuse (marked with white arrows), which also proves that MDs with low and medium DE values as wall materials have a poor protective effect on flavor substances during storage.

Of the samples containing MDs with different DE values, P_1_ at a high RH had the strongest hygroscopicity, which led to the wall material being more vulnerable to damage and the greatest release of flavor. During the whole storage process, the hydrophobic structure formed by MD with a high DE value combined with oil droplets of EA more closely, and thus the aggregation and diffusion of droplets in P_3_ were significantly improved. Previous studies have shown that when the degree of polymerization of the wall material is high the interaction between the flavor substance and the wall material is reduced, which results in a low retention rate of the flavor substance. Similar results were observed in the case of P_2_. Although the hygroscopicity was the lowest, owing to the weak interaction between the wall material and the core material the phenomena of oil droplet aggregation and outward diffusion were most obvious [31].

### 3.5. Release of Flavor during the Rehydration of Microcapsules

#### 3.5.1. Dynamic Dissolution of Microcapsules

**Figure 8:**
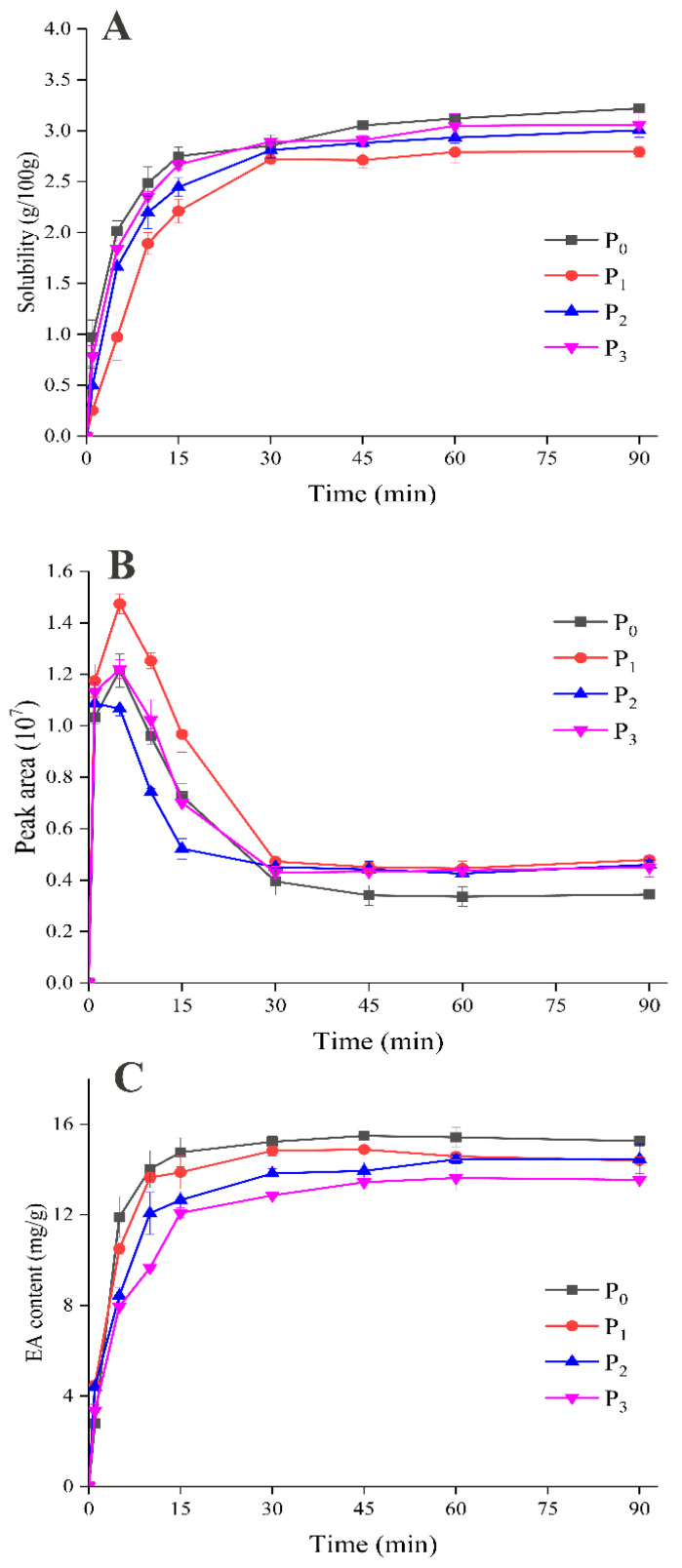
Changes of dynamic dissolution, Ethyl acetate (EA) release and content of EA content in liquid phase after 90 min of dissolution of powdered microcapsules. ((**A**) Solubility, (**B**) EA release, (**C**) EA content).

The dynamic dissolution of microcapsules is shown in Figure 8A. The results show that the dissolution rate of the powders was fast within 0–5 min after dissolution began, then increased slowly, and finally tended to become slow. After dissolution for 90 min, the sample with WPI alone as the wall material had the highest solubility, whereas the solubility of the samples to which MD was added gradually increased as the DE value increased. Generally speaking, a higher DE value corresponds to a greater degree of hydrolysis of MD, shorter molecular chains, and a corresponding increase in hydrophilic groups, which thus increases the solubility of powder microcapsules [32]. Table 2 shows the basic physical characteristics of the liquid emulsion before spray drying and the reconstructed emulsion after dissolution. The average droplet size of the liquid emulsion was between 247 and 290 nm, with a single-peak distribution. The increase in droplet size after the addition of MD was due to an increase in the viscosity of the emulsion. The zeta-potential and particle size of the reconstructed emulsion were similar to those of the liquid emulsion. It is believed that after the dissolution of the powder microcapsule an emulsion is formed in the closed system of core material, wall material, and water, and the wall material can still capture flavor substances via hydrophobic interactions in the reconstructed emulsion. Thus, the distribution of flavor substances will change in this closed environment.

#### 3.5.2. Retention and Release of EA during Dissolution of Microcapsules

In this case, it is interesting to see from Figure 8B that the general trend in the release of EA during dissolution was a rapid increase at first, which then slowed down to reach a final equilibrium. In comparison with P_0_, although P_1_ had comparatively poor solubility, a large amount of EA was still released at the beginning of dissolution owing to the high loading of the flavor substance. In addition, after dissolution for 90 min, more EA escaped from the samples in which MD was used as the wall material, and P_3_ exhibited the lowest retention of EA.

This phenomenon contributed to an understanding of the effect of powder microcapsules on the release of flavor substances in the dissolution process. At the initial stage of dissolution (0–5 min), the wall material was destroyed when the powder was exposed to water. Most of the EA was released to the headspace air rapidly, and the rest remained in the newly formed emulsion after dissolution. When the dissolution process reached the middle stage (5–30 min), the reconstructed emulsion gradually formed and the excess EA in the headspace air was recaptured until EA reached a new gas–liquid equilibrium in the bottle. Therefore, the headspace concentration of EA remained stable at a low level after dissolution for 30 min. The content of EA in the reconstituted emulsion is shown in Figure 8C and corresponded to the headspace concentration. The EA content increased first and then tended to be stable throughout the whole dissolution process, which was contrary to the trend in the release of EA. At the beginning of the dissolution process, namely, from 0 to 30 min, with the destruction of the wall materials unreleased EA would gradually participate in the formation of the reconstituted emulsion. When the whole system reached a dynamic equilibrium, the EA content tended to reach a balance and eventually remained unchanged, which confirms our conjecture.

#### 3.5.3. Mechanism of Release of Flavor Substances during Dissolution of Powder Microcapsules

**Figure 9:**
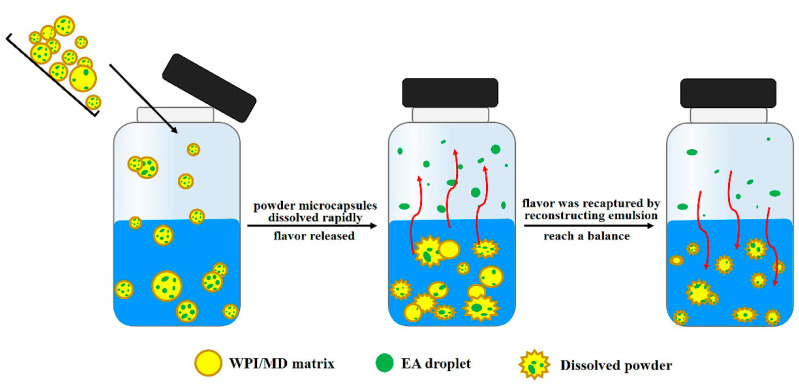
Schematic diagram of ethyl acetate (EA droplet) release when the powdered microcapsules using the wall materials whey protein and maltodextrin (WPI/MD matrix) are dissolved.

The dissolution of powder is a prerequisite for achieving the functionalities of microcapsules in a real food system. On the basis of the experimental results, we have established the mechanism of flavor release when the microcapsules are dissolved, as shown in Figure 9. When the microcapsules were dissolving, the wall materials were progressively eroded and the physical structures of the powder were also destroyed. At the same time, the core flavor substance was believed to escape from the solid matrix and be released into the surrounding solution or headspace air. In powder microcapsules, increasing the solubility of the wall materials can help to release a large amount of flavor substances in the dissolution process. With the increase of dissolution time, water and residual powder can form an emulsion again, which can recapture the flavor released by the interaction between the wall material and the flavor substance. After the formation of the reconstituted emulsion, the distribution coefficient of the whole system increases, and finally achieve a dynamic equilibrium in the closed system by recapturing the flavor substances.

## 4. Conclusions

In this study, MD/whey protein encapsulation systems with different DE values were investigated. The four samples could effectively encapsulate EA and improve its storage stability. MDs with high DE values had a stronger binding ability with the core material, which more effectively delayed the release of EA during storage. In addition, during the dissolution of microcapsules, high DE values also helped to release more EA. This proves that using WPI as the main substrate with a certain amount of added MD with a high DE value as the wall material can not only provide better protection for flavor substances during storage but also increase the release of flavor in the dissolution process. This finding will help us to understand and explain the effect of dynamic flavor release from powder microcapsules during storage and dissolution and provide more ideas for the design of powder microcapsules. For example, powder microcapsules are used in fruit and vegetable juices, yogurts, and functional drinks to enhance the flavor quality and improve the functional value of such products. 

## Figures and Tables

**Table 1:** Physical properties of powdered microcapsules.

Sample	Yield (%)	Moisture Content (%)	Particle Size (μm)	Encapsulation Efficiency (%)	Loading (mg/g)	Bulk Density (g/cm^3^)	Tap Density (g/cm^3^)	HR	CI (%)
P_0_	85.7 ± 1.2 ^c^	4.8 ± 0.1 ^a^	9.2 ± 3.1 ^a^	73.5 ± 0.7 ^a^	19.8 ± 2.5 ^a^	0.1655 ± 0.0019 ^a^	0.2263 ± 0.0124 ^a^	1.41 ± 0.07 ^a^	29.1 ± 3.4 ^a^
P_1_	70.4 ± 3.4 ^a^	4.4 ± 0.1 ^b^	9.9 ± 3.3 ^a^	70.7 ± 4.5 ^a^	18.3 ± 0.1 ^a,b^	0.1527 ± 0.0038 ^b^	0.2223 ± 0.0049 ^a^	1.46 ± 0.06 ^b^	31.3 ± 2.7 ^b^
P_2_	75.3 ± 1.1 ^b^	4.6 ± 0.1 ^c^	9.8 ± 2.8 ^a^	73.9 ± 1.7 ^a^	16.5 ± 1.0 ^b,c^	0.1539 ± 0.0029 ^b^	0.2313 ± 0.0113 ^a^	1.51 ± 0.09 ^b^	33.5 ± 3.9 ^b^
P_3_	82.9 ± 0.9 ^c^	4.2 ± 0.1 ^d^	8.9 ± 2.9 ^a^	84.2 ± 1.2 ^b^	13.9 ± 1.2 ^c^	0.1560 ± 0.0006 ^b^	0.2333 ± 0.0159 ^a^	1.58 ± 0.09 ^b^	36.6 ± 3.5 ^b^

^a–d^ Different letters in the same column indicate significant differences (*p* < 0.05).

**Table 2:** Properties of emulsion and reconstructing emulsion.

Sample	Emulsion		Reconstructing Emulsion	
	Zeta Potential (mV)	Droplet Size (nm)	Zeta Potential (mV)	Droplet Size (nm)
P_0_	−27.8 ± 0.5 ^ab^	264.9 ± 7.9 ^ab^	−28.6 ± 0.4 ^a^	247.7 ± 10.9 ^a^
P_1_	−29.8 ± 0.6 ^c^	290.0 ± 13.4 ^c^	−28.1 ± 0.7 ^a^	248.0 ± 1.4 ^a^
P_2_	−27.2 ± 1.6 ^ab^	247.8 ± 2.7 ^a^	−28.9 ± 0.6 ^a^	300.7 ± 9.2 ^b^
P_3_	−26.7 ± 1.3 ^a^	275.2 ± 5.8 ^bc^	−28.5 ± 1.4 ^a^	241.3 ± 4.9 ^a^

^a–c^ Different letters in the same column indicate significant differences (*p* < 0.05).
